# Heteromorphism of stamens in monoclinous flowers of *Tinantia erecta* (Jacq.) Fenzl as an example of high variability of the androecium in the Commelinaceae family

**DOI:** 10.1007/s00709-020-01522-z

**Published:** 2020-06-26

**Authors:** D. Tchórzewska, R. Marciniec, K. Deryło, K. Winiarczyk

**Affiliations:** 1 Department of Cell Biology, Maria Curie-Skłodowska University, Lublin, Poland; 2 Department of Molecular Biology, Maria Curie-Skłodowska University, Lublin, Poland

**Keywords:** Commelinaceae, *Tinantia erecta*, Heteromorphism of stamens, Microsporogenesis, Microtubular cytoskeleton

## Abstract

Representatives of the family Commelinaceae are characterised by morphologically, anatomically, or functionally diverse stamens (common presence of staminodia), which produce diverse pollen grains. The heteromorphism of stamens noted in all Commelinaceae species is a particular example of the evolutionary modification of the androecium in entomophilous plants. The morphological, anatomical, and cytological analyses of the androecium as well as the analysis of the microsporogenesis process and the formation of the male gametophyte in *Tinantia erecta* (a species belonging to the family Commelinaceae) have demonstrated that the morphologically diverse stamens in this species do not differ anatomically. Furthermore, the process of microsporogenesis followed by gametogenesis occurring in the stamens yields pollen grains with the same morphology, cytology, and function. Therefore, despite the large morphological diversity of the androecium, all anthers in *T. erecta* produce male gametophytes that are identical in every respect, which is a unique feature in species from the Commelinaceae family. Additionally, *T. erecta* is capable of self-pollination; hence, it can be claimed that the species uses its entire reproductive potential to produce seeds and a next generation.

## Introduction

In production of seeds in closed ovaries and specialisation of broad-sense sexual reproduction processes, angiosperms have won the competition with gymnosperms and dominated the contemporary flora. The dominance was facilitated by the more effective pollination and development of adaptations to cross-pollination, which ensures variability of forms and further evolutionary potential (Johri and Ambegaokar 1984; Wyatt 2012).

The flower of angiosperms, i.e. a specialised sexual reproduction organ, is structured according to a uniform design but exhibits huge morphological diversity. There are many types of flowers in nature, and this organ can be systematised according to various criteria, e.g. the sexual system, perianth shape, availability of nectaries, pollination modes, and type of the pollen vector. As far as the type of the pollen vector is concerned, even within one family, e.g. Commelinaceae, where insects are pollen vectors, species may be divided into those with a specialised pollinator and those pollinated by many insect species. Observations conducted in natural conditions indicate that plants of the Commelinaceae family are most frequently visited by various species of bees and flies from the Bombyliidae and Syrphidae families (Faden 1992). Faden (1992) reported frequent visits by other insects from the following orders: Diptera, Coleoptera (beetles from the families Melyridae, Buprestidae, Mordellidae), Hemiptera (from the suborder Homoptera), Orthoptera, and Thysanoptera, as well as occasional visits by ants (from the order Hymenoptera). Additionally, as demonstrated by Schuster and Schuster (1971), various butterfly species were attracted to *Tripogandra cumanensis* flowers. Based on these observations, it was found that most species of the Commelinaceae family do not have specialised pollinators. An exception is two species of the genus *Dichorisandra*, which have a specialised pollinating insect (Sigrist and Sazima 1991). In turn, *Callisia repens* is the only wind-pollinated species of the Commelinaceae family (Faden 1992). It should be emphasised that numerous modifications of flowers have enabled plants to adapt perfectly to particular environments, especially effective pollination allowing the most favourable fertilisation, contributing to the huge reproductive success of flowering plants (Johri and Ambegaokar 1984; Friedman and Williams 2004).

The Commelinaceae family comprises plant species characterised by high morphological diversity, which makes it very interesting from an embryological, biogeographic, evolutionary, and systematic point of view. This family includes 41 genera represented by 650 plant species, and their high morphological diversity is the cause of difficulties in establishment of a clear taxonomic classification of its representatives (Faden and Hunt 1991; Faden 1998; Pellegrini 2017). Molecular phylogenetic methods have allowed revision of the current systematics of the studied family (Faden et al. 2009). The present classification is based not only on the analysis of morphological traits but also on the analysis of the chloroplast gene rbcL (Evans et al. 2000, 2003; Burns et al. 2011).

Despite the high morphological diversity of flowers of representatives of the Commelinaceae family, some traits are typical of all species. For example, the flowers are bisexual, the anthesis stage lasts only 1 day, all generative floral elements develop almost simultaneously, and trichomes are commonly present in the flowers (Faden 2000; Hardy and Stevenson 2000a, b). In addition, a characteristic trait in Commelinaceae species is the presence of a morphologically, anatomically, or functionally diverse androecium (common presence of staminodia), in which diverse pollen grains are produced (Vogel 1978; Faden 1992; Hrycan and Davis 2005).

The investigations presented in this study were carried out on a monocotyledonous plant *Tinantia erecta* (Jacq.) Fenzl, which is a poorly explored species from the Commelinaceae family. The monoclinous flowers of this species exhibit general features of the family: the anthesis lasts 1 day, the generative floral elements mature almost simultaneously, and there are abundant trichomes and diverse androecia. The present study was focused on comprehensive investigations of the androecium, based on morphological, anatomical, and cytological analyses as well as exploration of the processes of microsporogenesis and formation of the male gametophyte in *T. erecta*. To date, many reproductive strategies and androecium types have been described in Commelinaceae species (Walker-Larson and Harder 2000; Hrycan and Davis 2005; Pellegrini 2017), but there are no reports on the role of stamens in *T. erecta*. As demonstrated by the comprehensive morphological, anatomical, cytological, and functional analyses of pollen produced by *T. erecta*, the species has stamens with the same anatomy, cytology, and function despite their huge morphological diversity. This study extends the knowledge of the flowering biology of *T. erecta* with reference to the diverse reproductive strategies in the Commelinaceae family, which is an attempt to explore the trend in adaptive changes in this family.

## Materials and methods

### Plant material

*T. erecta* plants were grown in the greenhouse of Maria Curie-Sklodowska University at 23 °C on a universal, slightly acid soil (pH 5.5–6.5) under a natural photoperiod depending on the season. Seeds were obtained from Maria Curie-Sklodowska University Botanical Garden (Poland), where the species was deposited after being brought from the Botanical Garden of the Technical University of Dresden (Germany)-catalogue number MX-0-DR-003782. Fifty flowers were selected randomly from approximately 70 plants for all analyses.

Macroscopic photographs of the plants were taken with a Nikon D300 camera equipped with an AF MICRO NIKKOR 60-mm optical lens.

Macroscopic images of single flowers and floral elements were taken in an Olympus SZX16 stereoscopic microscope equipped with a DP 72 photographic camera.

### Light microscopy

For histological observations, isolated *T. erecta* anthers were fixed in a mixture of 2.5% paraformaldehyde and 2.5% glutaraldehyde in cacodylate buffer (pH 7.2) for 24 h at room temperature. The material was then rinsed in cacodylate buffer and placed in a 2% solution of osmium tetraoxide in deionised water. Next, it was dehydrated in a series of alcohol, placed in 100% ethanol, and embedded in LR White acrylic resin (Sigma). The material was cut into semi-thin sections (1 μm) using a Leica EM UC7 microtome (Wetzlar, Germany) and stained with a 1% toluidine blue solution. The sections were prepared under a light microscope Nikon Eclipse Ni-U with a digital camera and NIS-Elements BP software.

Crushed preparations were made for cytological observations. Differently sized anthers of *T. erecta* were crushed and stained with acetocarmine (Gerlach 1977). The observations were carried out under a light microscope Nikon Eclipse Ni with Nomarski contrast. Photographic documentation was made with a digital camera and NIS-Elements BP software.

### Immunofluorescence method

Pieces of differently sized *T. erecta* anthers were fixed for 24 h in 4% paraformaldehyde and 0.25% glutaraldehyde in MT stabilising buffer (MSB) (Baluska and Barlow 1993), pH 7.0, at room temperature. They were then rinsed in MSB buffer, dehydrated, embedded in polyethylene glycol, and sectioned according to the method proposed by van Lammeren et al. (1985) modified by Tchórzewska et al. (2008). Two-micrometre-thick sections were mounted on slides coated with 2% organosilan (Sigma) and the slides were rinsed 3 times for 5 min each in phosphate-buffered saline (PBS). Next, they were treated with 0.1 M NH4Cl in PBS, washed twice for 5 min in PBS, and blocked with 0.1% bovine serum albumin (BSA) in PBS for 30 min. Subsequently, the slides were incubated in a moist chamber for 60 min at 37 °C with monoclonal anti-mouse β-tubulin (Sigma) diluted 1:200 in 0.1% BSA in PBS. After washing with 0.1% BSA in PBS (3 times for 15 min), incubation with a secondary antibody was carried out for 60 min at 37 °C. The secondary antibody conjugated with fluorescein isothiocyanate (Sigma) was diluted 1:200 in PBS with 0.1% BSA. 4′,6-diamidino-2-phenylindole dihydrochloride (DAPI) was added to the sections to stain DNA in the nuclei and organelles. Laser scanning confocal microscope LSM780 Zeiss equipped with a Plant Apochromat 63x/1.40 Oil DIC M27 objective was used for imaging the anther sections. Two-channel imaging was performed using a 405-nm diode laser for DAPI and a 488-nm Argon laser for FITC. Fluorescence was recorded in the range of 410–460 nm and 500–560 nm, respectively. To avoid photobleaching, both lasers worked at 2% power.

### Scanning electron microscope

Samples for observation in SEM were prepared according to the method developed by Talbot and White (2013). Fresh material was fixed by immersion in methanol for 10 min and then rinsed in ethanol 2 × 30 min. Next, the material was dried in a CO2 atmosphere, sputter coated with gold, and viewed in SEM LEO1430VP with 15 kV acceleration potential. The documentation was made using the INCA Mapping software (Billerica, MA, USA).

### Pollen viability

*T. erecta* pollen grains were collected immediately after anthesis and stained with the Alexander assay according to the method proposed by Peterson et al. (2010). In living pollen grains, the protoplast stained purple and the cell wall stained green. Dead pollen grains stained only green. The preparations were observed under a light microscope Nikon Eclipse Ni-U with a digital camera and NIS-Elements BP software.

### In vivo germination of pollen grains

To test the in vivo germination capacity, the pollen grains were placed on the stigma. The analyses were performed on 10 randomly chosen pistils in three replicates. After 30 min, the styles were sampled and placed in a 0.1% solution of aniline blue for 30 min. Next, the styles with stigmas were delicately crushed on glass slides, and germinating pollen grains were observed under a Nikon Eclipse Ni fluorescence microscope using a 330- to 380-nm excitation filter and a 420-nm cut-off filter (Tchórzewska et al. 2015). In order to calculate the per cent of germinating pollen grains, pollen with pollen tubes penetrating stigma cells or style tissue were analysed under a light microscope. Photographic documentation was made with a digital camera and NIS-Elements BP software.

### Self-pollination experiment

The experiment was carried out in a greenhouse on randomly selected plants growing at least 50 cm apart. All anthers were emasculated from the flower buds of *T. erecta*. Next, pollen grains from their own anthers or from another flower located on the same or another plant were transferred to the pistil stigma using a brush. Each variant was prepared in five replicates. Pollinated flowers were insulated to protect against uncontrolled transfer of pollen grains. After approximately 30 days, the number of seeds formed on plants pollinated with own and foreign pollen was assessed.

## Results

### Morphological analyses of the flower

**Fig. 1:**
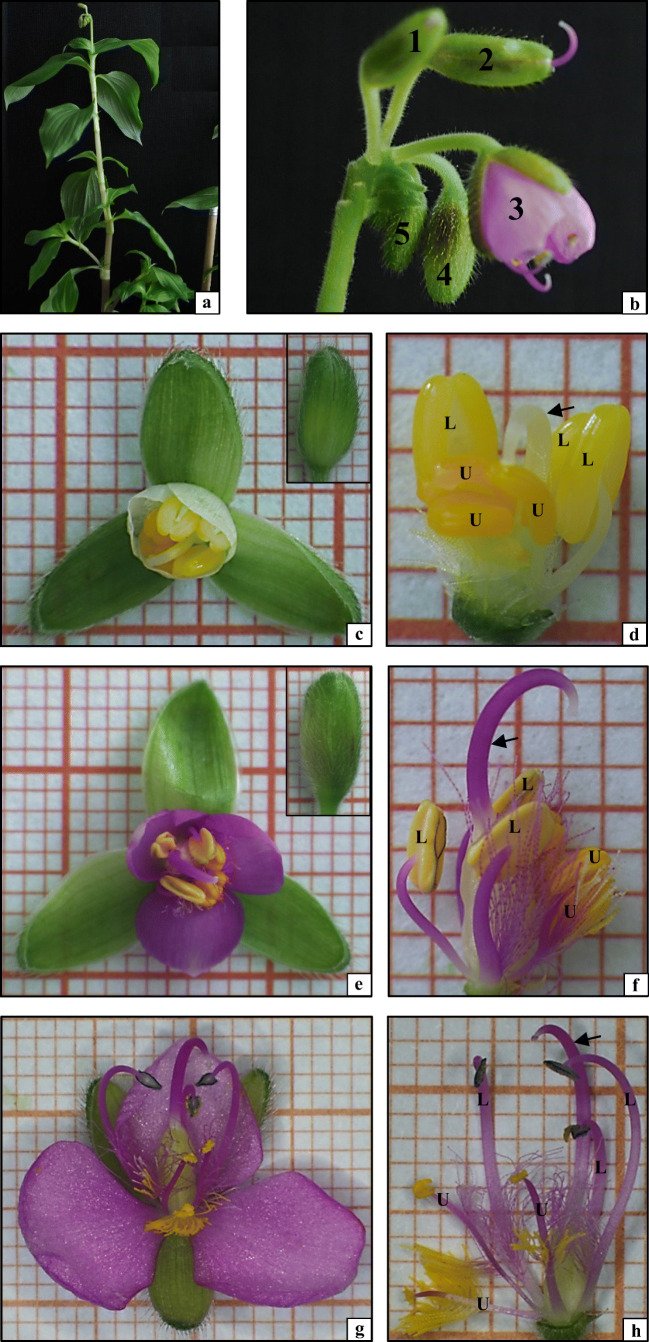
Morphology of *Tinantia erecta*: **a** aboveground shoot; **b** inflorescence (flower buds: 1, oldest; 5, youngest); **c** elements of a flower from a 5-mm-long bud; **d** generative elements of a 5-mm flower bud; **e** elements of a flower from a 10-mm-long bud; **f** generative elements of a 10-mm flower bud; **g** flower in the anthesis stage; **h** generative elements of a flower in the anthesis stage (U, upper stamens; L, lower stamens; arrow, pistil style)

The *Tinantia erecta* species analysed in the present study had fleshy aboveground shoots with numerous monopodial branches. The stems had nodes and short internodes with alternately arranged leaves. All the leaves were sessile, had no petioles, and their lower part formed a sheath around the stem. The simple undifferentiated *T. erecta* leaves exhibited an entire margin, parallel venation, green colour, and elliptical shape. The height of aboveground shoots at the time of flowering was 60–80 cm (Fig. 1a).

**Fig. 2:**
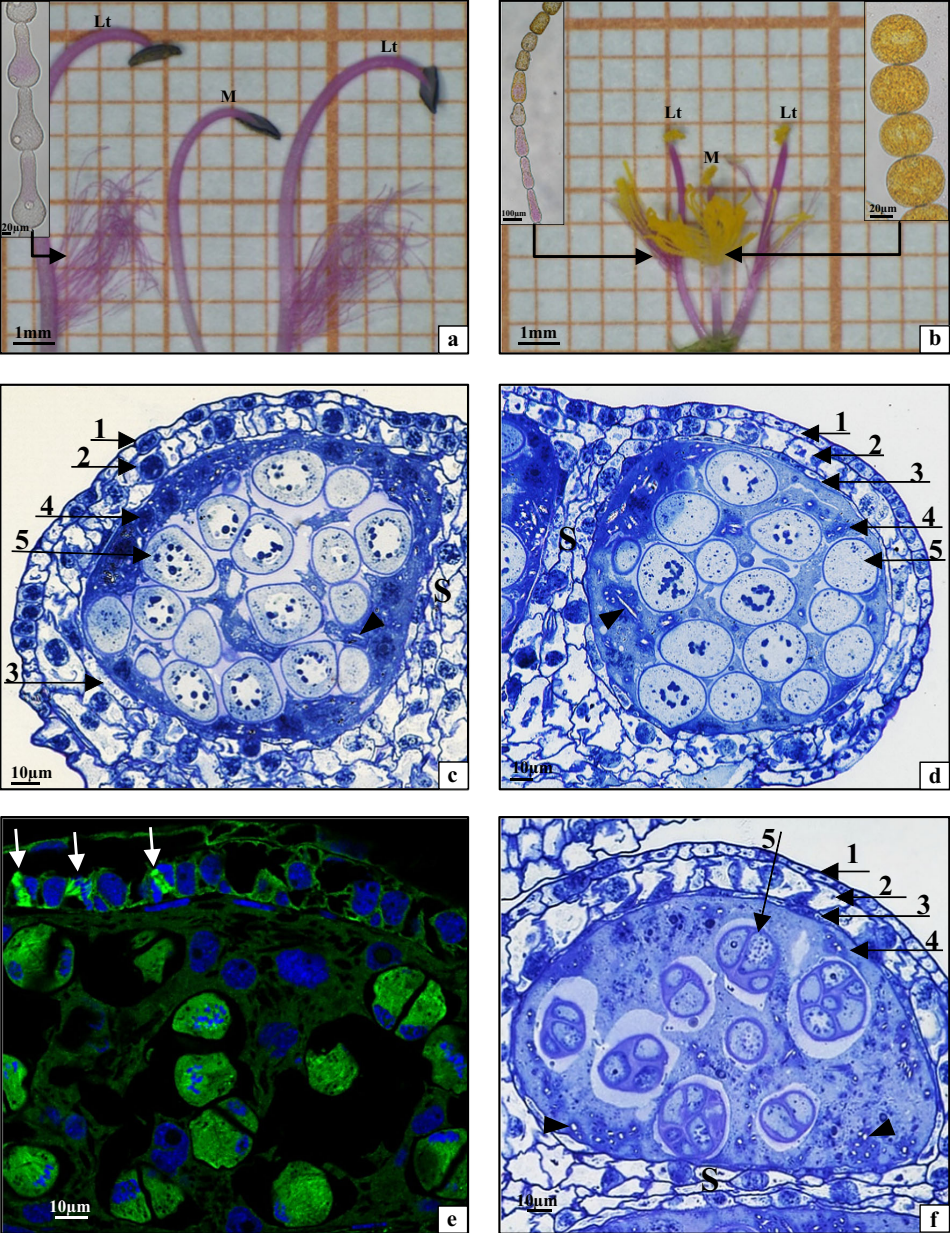
Stamens from a flower in the anthesis stage: **a** stamens from the lower whorl; **b** stamens from the upper whorl (Lt, lateral stamens; M, middle stamen; small pictures, trichomes). Cross sections through pollen sacs: **c** upper lateral stamen (ULt), early prophase I; **d** lower lateral stamen (LLt), late prophase I; **e** upper middle stamen (UM), meiotic division II; **f** lower middle stamen (LM), microspore tetrads. **c**, **d**, **f**: 1, epidermis; 2, endothecium; 3, middle layer; 4, tapetum; 5, meiocytes; S, septum; arrowhead, calcium oxalate crystals; toluidine blue staining. **e** arrows, configurations of the mictotubules during mitosis in endothecium cells; MTs visualised by indirect immunofluorescence (green colour), nuclear DNA stained with DAPI (blue colour)

The *T. erecta* plants had a cymose inflorescence consisting of 5 to 7 different ages and respectively different sized flower buds covered by abundant glandular trichomes. Immediately before the anthesis, the oldest and the youngest buds in a single inflorescence were 10–12 mm and 2–1.5 mm long, respectively (Fig. 1b). Only the oldest bud at the top of the inflorescence was opened during the day for 7–8 h. The *T. erecta* flowers were small, monoclinous, with a tripartite structure. They exhibited varied symmetry: an actinomporphic perianth and zygomorphic generative elements. The calyx was composed of three navicular sepals, and the corolla had three undifferentiated purple petals. The centre of the flower was occupied by a tripartite superior pistil, which consisted of an ovary, a long, curved, purple style, and a small stigma. The pistil was surrounded by six stamens in two whorls (three stamens in each whorl) located in the upper and lower parts of the receptacle (Fig. 1c–h). The stamens were morphologically diverse already at the beginning of development (Fig. 1d). The largest differences in the morphological structure of the androecium were visible in the flower anthesis stage (Fig. 1g, h). The upper (U) and lower (L) stamens differed in their size, the presence and colour of trichomes, and the colour of mature anthers (Figs. 1h and 2a, b). Additionally, the stamens within the upper and lower whorls were differentiated as well. The androecium whorl located in the lower part of the receptacle was composed of two lateral stamens (Lt) and one middle stamen (M) (Fig. 2a). Their elongated anthers were light yellow at the beginning of development (Fig. 1d, f) and dark purple in the anthesis stage (Figs. 1h and 2a). The lateral stamen filaments (Fig. 2a, Lt) were long (8–10 mm), higher than the stigma of pistil, and bent towards the stigma. At the base, the filaments were covered by clavate purple trichomes (Fig. 2a, small picture). In turn, the middle stamen (Fig. 2a, M) had a slightly shorter filament (6–8 mm) devoid of trichomes (Fig. 2a). The androecium whorl located in the upper part of the receptacle had two lateral stamens (Lt) and one middle stamen (M) (Fig. 2b). However, the upper stamens (U) differed in all other traits from the lower ones (L). The anthers of the upper stamens were yellow from the beginning of their development to the anthesis stage (Fig. 1d–h). Their filaments were erect and short (5–7 mm) and had trichomes halfway along their length (Fig. 2b). The trichomes of the lateral stamens were built of rod-shaped purple cells at the filament, while the apical parts of these trichomes were formed of oval yellow cells (Fig. 2b, Lt, small picture). The middle stamen in the upper whorl differed from the lateral stamens: it was shorter (5–6 mm) and had numerous trichomes composed of large oval yellow cells (Fig. 2b, M, small picture).

Therefore, it can be concluded that the elements of the *T. erecta* flower, especially the androecium, are characterised by a highly diverse morphological structure.

### Anatomical analyses of the androecium

To determine whether the large morphological diversity observed between *T. erecta* anthers located in the upper and lower part of the receptacle is reflected in anatomical differences between these structures, the anatomy of lower lateral (LLt, Fig. 2c) and lower middle anthers (LM, Fig. 2e) as well as upper lateral (ULt, Fig. 2d) and upper middle anthers (UM, Fig. 2f) was analysed. It was found that the heads of all types of anthers consisted of four pollen sacks (microsporangia) linked by a connective tissue. The wall of these anthers was composed of the following layers: the epidermis, endothecium, middle layer, and tapetum, which surrounded the pollen mother cells PMC and filled the anther loculus. The anther loculi were separated from each other by a transverse septum (S) composed of several layers of irregular spindle-shaped cells. During microsporogenesis, the cells of the middle layer in the anther wall were flattened or disappeared due to the gradual development of microsporocytes (Fig. 2c–f). In contrast, the endothecium cells underwent anticlinal divisions and enlarged (Fig. 2e). The tapetum in all the upper and lower *T. erecta* anthers represented the ameboid type; hence, the cells of this tissue visible in early prophase I (Fig. 2c) gradually lost their integrity and transformed into periplasmodium surrounding meiotically dividing PMCs (Fig. 2d–f). A distinctive feature was the presence of numerous single needle-shaped CaOx crystals (raphides) in the loculi of all anthers throughout the microsporogenesis period (Fig. 2c–f, arrowhead).

The analyses demonstrated that the upper lateral (ULt) and middle (UM) anthers had the same anatomical and cytological structure as the anthers of the lower stamens (LLt and LM).

### Analyses of microsporogenesis and microgametogenesis

**Fig. 3:**
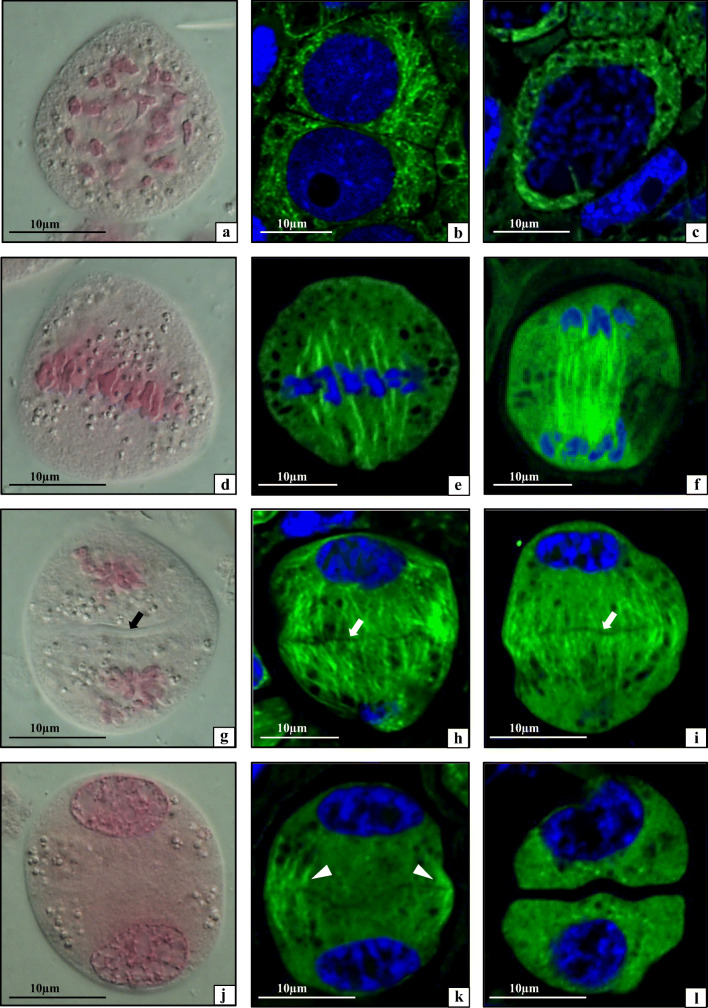
Microsporocytes of *Tinantia erecta* during meiosis: **a–c** prophase I; **d**, **e** metaphase I; **f** anaphase I; **g** early telophase I; **h–k** telophase I; **l** prophase II; arrows, primary septum; arrowheads, MTs of a disappearing phragmoplast. **a**, **d**, **g**, **j** Images from LM with Nomarski contrast. **b**, **c**, **e**, **f**, **h**, **i**, **k** MTs visualised by indirect immunofluorescence (green colour), nuclear DNA stained with DAPI (blue colour)

**Fig. 4:**
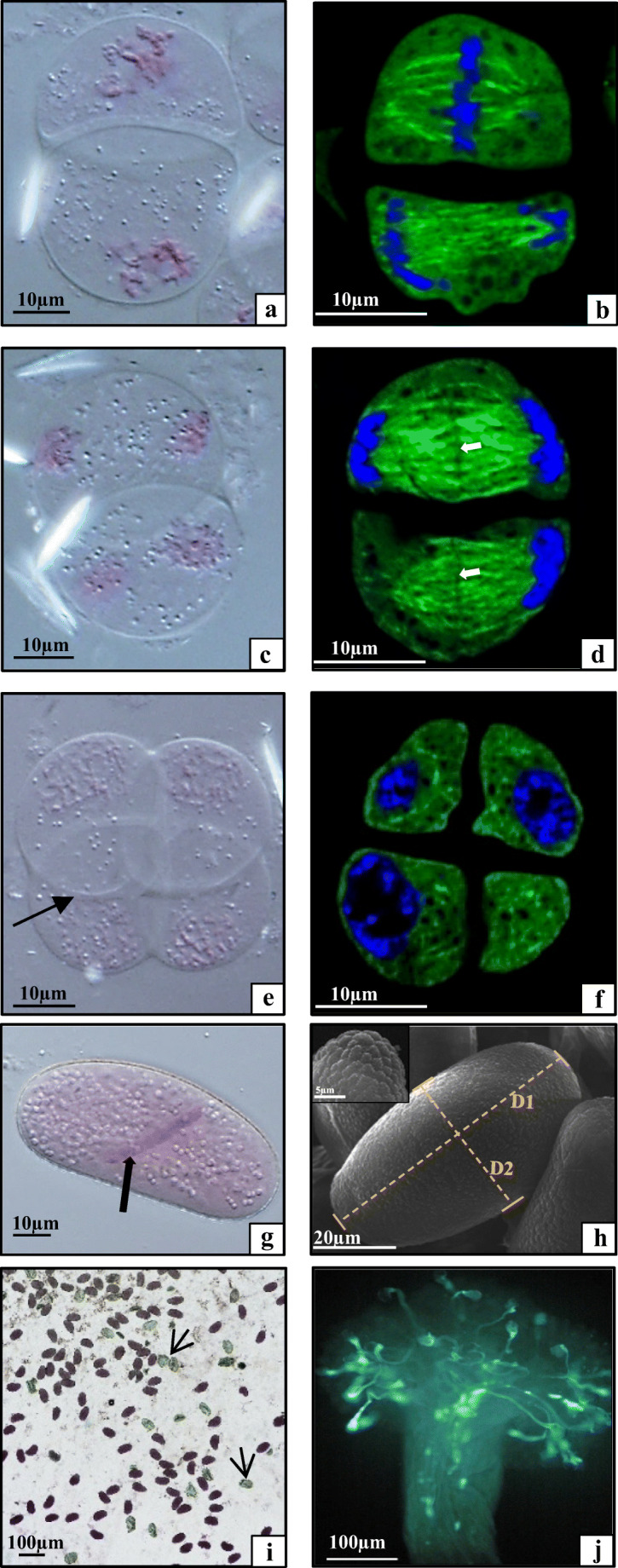
**a**–**d** Microsporocytes of *Tinantia erecta* during meiosis, **a** metaphase II; **b** late metaphase II; **c**, **d** anaphase II, arrow, primary septum. **e**, **f** Microspore tetrad, arrow, callose wall. **g** Two-celled pollen grain, arrow, generative cells. **h** Pollen grain viewed in SEM, D1, long axis = 65.22 μm; D2, short axis = 35.36 μm; small picture, sculpture of the pollen grain wall. **i** Pollen grain stained with the Alexander assay, viable pollen grains stained purple, dead pollen grains stained green (arrow). **j** Germination of pollen grains on the stigma, visible fluorescence of the callose wall of the pollen tube stained with aniline blue

The anther loculus is a site of microsporogenesis, which results in the formation of male gametophytes, i.e. pollen grains from pollen mother cell (PMC). All stages of microsporogenesis occurring in the ULt, UM, LLt, and LM anthers were analysed in the *T. erecta* in this study. Cytological analyses (light microscopy) and immunocytochemical assays (fluorescence microscopy) of meiotically dividing cells were carried out. They demonstrated both the nuclear division and the organisation of cellular structures (plastids, mitochondria, and tubulin cytoskeleton) at each stage of microsporogenesis (Figs. 3a–l and 4a–f). At the beginning of meiosis, in prophase I, the microsporocytes in all four types of anthers of the examined species had a centrally located large nucleus. Their cell organelles (mainly plastids and mitochondria) were dispersed evenly in the cytoplasm. Condensation of nuclear chromatin and formation of chromosomes was observed in the nucleus (Fig. 3a). The tubulin cytoskeleton in prophase I was composed of a well-organised dense network of microtubules (MTs) dispersed evenly in the cytoplasm (Fig. 3b, c). In metaphase I, a normally formed metaphase chromosome plate surrounded by cell organelles evenly distributed in the cytoplasm was visible in the meiocytes in all upper and lower anthers (Fig. 3d). The tubulin cytoskeleton in such cells formed a karyokinetic spindle consisting of numerous MT filaments (Fig. 3e). Next, in anaphase meiocytes, polar microtubules of the karyokinetic spindle with characteristic parallel arrangement were visible between chromosomes present on the opposite poles of the cell (Fig. 3f). The meiotic telophase I cells of all four types of anthers exhibited decondensation of chromosomes and formation of daughter nuclei with cell organelles clustered in the space between these nuclei. The primary septum, i.e. the first stage of formation of the primary wall, was visible in the equatorial plane of the cell (Fig. 3g, arrow). In such cells, there was also a well-developed phragmoplast consisting of numerous MTs extending from the nuclei to the equatorial plane of the cell (Fig. 3h, i). In telophase I, the meiocytes of all examined types of anthers had daughter nuclei with a visible nuclear envelope and cell organelles clustered in the equatorial plane at the cell periphery (Fig. 3j). The phragmoplast microtubules underwent depolymerisation and were only visible in the parietal cytoplasm (Fig. 3k). In all four types of anthers, successive cytokinesis occurred and the second meiotic division took place in a two-celled meiocyte, i.e. the dyad. In the *T. erecta* meiocytes, the phragmoplast completely disappeared in prophase II and tubulin filaments of the cytoskeleton were visible only near the nucleus (Fig. 3l). In metaphase II, two metaphase chromosome plates formed in the dyad, the cell organelles were dispersed evenly in the cytoplasm (Fig. 4a), and all MTs formed two karyokinetic spindles. In the cells of all types of the upper and lower anthers, asynchronous karyokinesis was observed in metaphase II, i.e. when metaphase occurred in one dyad cell, anaphase was already in progress in another cell (Fig. 4b). In the next stage of meiosis, i.e. anaphase II, there were groups of daughter chromosomes on the opposite poles of the dyad cells (Fig. 4c). Next, in early telophase II, an abundant phragmoplast was observed with a primary septum forming in the equatorial plane of the dyad cells (Fig. 4d). The microspore tetrads analysed in all four types of anthers were initially covered by a thin callose wall (Fig. 2e). The tubulin cytoskeleton in slightly older microspore tetrads formed numerous short sections scattered in the cytoplasm (Fig. 4f). After microsporogenesis, two-celled pollen grains composed of a vegetative and generative cell formed in the microgametogenesis process (Fig. 4g). In all types of anthers, mature pollen grains had no pori, a single-colpus, an elongated shape (Fig. 4h, D1—long axis = 65.22 μm; D2—short axis = 35.36 μm). The surface of such pollen grains had verrucate sculpture with irregularly sized verrucae (Fig. 4h, small pictures).

To sum up, it can be concluded that normal karyokinesis, neutral-equatorial chondriokinesis, and successive cytokinesis occur during the microsporogenesis process in all anthers constituting the *T. erecta* androecium. These processes lead to the formation of morphologically undifferentiated pollen grains.

### Viability and germination of pollen grains

The next step of the investigations consisted in comparative analyses of the viability of pollen grains formed in the ULt, UM, LLt, and LM anthers. The Alexander assay showed 80–85% viability of pollen grains in all types of anthers. In this test, viable pollen grains were stained purple (protoplast) and green (cell wall). Dead pollen grains were completely green (Fig. 4i, arrows). Additionally, to assess the ability of the pollen grains to germinate, in vivo analyses (on the stigma) were performed. Aniline blue staining showed the callose walls of the pollen tubes germinating on the stigma and penetrating the style. A germination rate of 40–50% of the *T. erecta* pollen grains was demonstrated (Fig. 4j).

As shown by the analyses, it can be concluded that all types of anthers in *T. erecta* flowers produce pollen grains characterised by the same level of viability and germinability.

### Self-pollination experiment

To check whether self-pollination is possible in *T. erecta*, we carried out an experiment in which emasculated flowers were pollinated with own or foreign pollen. Such flowers were protected against uncontrolled transfer of pollen grains. It was observed that seeds were set at a level of approximately 30–40% in the pollinated plants after both self-pollination and cross-pollination. Therefore, based on this experiment, it can be concluded that both self-pollination and cross-pollination are possible in *T. erecta*.

## Discussion

In the Commelinaceae family, although insects are the major pollen vectors, the flowers in these plants have a short anthesis stage and no nectaries, which is a limitation to the pollination process. Therefore, these species have developed numerous strategies to increase pollination success. The strategies are mainly associated with the visual aspect, which is attractive to insects, e.g. a distinct colourful perianth composed of different coloured corolla petals and sometimes colourful sepals, as in *Tinantia pringlei* and flowers of *Aneilema* and *Tradescantia* species (Hardy and Ryndock 2012). In addition to the multicoloured flowers, some elements of shoots are brightly coloured in some species (e.g. *Coleotrype madagascarica*) (Faden 1992). *T. erecta* belonging to the family Commelinaceae has flowers with a distinct colourful perianth and generative elements. It exhibits the general features of the family: the anthesis lasts 1 day, the generative floral elements mature almost simultaneously, and there are abundant trichomes and diverse androecia. However, there are no reports on the role of heteromorphic stamens in *T. erecta*; hence, the present study is focused on comprehensive structural and functional investigations of the androecium in this species.

The Commelinaceae plants have a large number of stamen structures that are especially attractive to potential pollinators. These include filament trichomes, anthers with pollen, or anther-like structures (staminodia). All these structures are morphologically and often functionally diverse within the flower. For instance, trichomes, which are characteristic of all species from the Commelinaceae family, have many shapes and colours. *Tinantia erecta* analysed in this study also has abundant morphologically diverse trichomes present on filaments. As we have shown, the trichomes are represented by three different types: trichomes composed of yellow round cells, yellow and purple trichomes with elongated cells, and purple trichomes built of bottle-shaped cells. In Commelinaceae species, trichomes occur mainly on stamen filaments and on the surface of sepals (genus *Floscopa*), corolla petals (genera *Cochlostema* and *Geogenanthus*), and the surface of the ovary and stigma (genus *Cyanotis*). It is believed that such trichomes play an important role not only in the pollination process but also as protection against desiccation and insect foraging (Faden 1992, 1998). However, the yellow trichomes that are commonly found in flowers of *Tinantia*, *Cochhiostema*, and *Geogenanthus* species serve essentially as insect attractants (Vogel 1978).

Another attractant for insect pollinators are anthers, which generally attract insects with produced pollen grains—a reward for pollinators. Species from the Commelinaceae family exhibit a huge morphological diversity of anthers, which can be regarded as elements of mimicry increasing the visual attractiveness of the flower. It should be pointed out, the morphological analysis carried out in the present study has revealed that the stamens in the *T. erecta* flower are located in one upper and one lower whorls, which in turn are differentiated into lateral and middle ones, exhibiting general features of Commelinaceae from this respect. All stamens differ in their size as well as the location, colour, and shape of the trichomes, and the colour of mature anthers; therefore, they exhibit morphological diversity. Based on the available literature, it can be concluded that all Commelinaceae species described so far have morphologically diverse stamens (Table 1). The exceptions are only *Aneilema neocaledonicum* (Faden 1991), *Commelina diffusa* (Oziegbe et al. 2013), and *Murdannia vaginata* (Faden 2001). As suggested by other authors, the three upper anthers in the *Tinantia* genus are smaller and surrounded by yellow trichomes; they are referred to as ‘advertising stamens’. The lower stamens are larger, produce more pollen, and are neutral to insects in terms of colour (Vogel 1978). Simpson et al. (1986) demonstrated that the upper anthers and surrounding trichomes found in *Tinantia anomala* absorb UV light in contrast to the petals located behind them, which reflect UV radiation. Additionally, as emphasised by researchers, the differentiation into lower and upper anthers is highly important for insect-pollinated species. An insect visitor in the flower touches the upper anthers but its abdomen has contact with lower anthers (Lee 1961), which increases the chance of cross-pollination.

**Table 1:** Heteromorphism of the androecium in Commelinaceae species (**+,** present; **−,** absent; *nd*, no data)

Species or genus	Morphological heterogeneity of anthers	Functional heterogeneity of anthers (staminodia)	Morphological heterogeneity of pollen	Functional heterogeneity of pollen	Reference
*Aneilema calceolus*	+	+	+	nd	Faden 1991; Faden 2000
*Aneilema clarkei*	+	+	+	nd	Faden 1991; Faden 2000
*Aneilema hockii*	+	+	+	nd	Faden 1991
*Aneilema lamuense*	+	+	−	nd	Faden 1991
*Aneilema neocaledonicum*	−	+	nd	nd	Faden 1991
*Anthericopsis*	+	nd	nd	nd	Faden 1985
*Aploleia*	+	−	nd	nd	Faden 1985
*Buforrestia*	+	+	nd	nd	Faden 2000
*Cochliostema odoratissimum*	+	+	nd	nd	Hardy and Stevenson 2000a
*Commelina benghalensis*	nd	+	+	−	Faden 2000
*Commelina coelestis*	+	+	+	+	Hrycan and Davis 2005
*Commelina communis*	+	+	nd	nd	Faden 1992; Ushimaru et al. 2003
*Commelina dianthifolia*	+	+	+	+	Hrycan and Davis 2005
*Commelina diffusa*	−	+	nd	−	Oziegbe et al. 2013
*Commelina erecta*	+	+	+	−	McCollum et al. 1984; Oziegbe et al. 2013
*Commelina lagosensis*	+	+	nd	+	Oziegbe et al. 2013
*Commelinopsis*	+	nd	nd	nd	Faden 1985
*Dictyospermum*	+	+	nd	nd	Faden 1985
*Floscopa*	+	+	+	nd	Faden 2000; Faden 2001
*Geogenanthus*	+	+	nd	nd	Faden 2000
*Murdannia audreya*	+	+	−	−	Faden 2001
*Murdannia dimorphoides*	+	+	−	−	Faden 2001
*Murdannia striatipeta*	+	+	−	−	Faden 2001
*Murdannia vaginata*	−	−	nd	nd	Faden 2001
*Palisota*	+	+	−	−	Faden 1983, 1998
*Palisota hirsuta*	+	+	+	−	Faden 1992, 2000
*Phaeosphaerion*	+	nd	nd	nd	Faden 1985
*Plowmanianthus dressleri*	+	+	−	−	Hardy et al. 2004
*Plowmanianthus grandifolius*	+	+	−	−	Hardy et al. 2004
*Plowmanianthus panamensis*	+	+	−	−	Hardy et al. 2004
*Polyspatha*	+	nd	nd	nd	Faden 1985
*Rhopalephora*	+	nd	nd	nd	Faden 1985
*Tinantia anomala*	+	−	+	−	Gębura et al. 2014
*Tinantia erecta*	+	−	−	−	our observations
*Tinantia pringlei*	+	−	nd	−	Hardy and Ryndock 2012
*Tricarpelema*	+	+	nd	nd	Faden 2000
*Tripogandra errulate*	+	nd	+	−	Handlos 1970, 1975; Faden 2000
*Tripogandra diuretica*	+	nd	+	+	Gamerro 1986; Faden 2000
*Tripogandra grandiflora*	+	nd	+	+	Lee 1961; Faden 1998; Faden 2000

It should be underlined that morphological diversity of the androecium described above is usually reflected in the anatomical and functional diversity (Table 1). However, it should be emphasised, that this is not the case of *T. erecta*, because the anatomical and cytological analyses of upper (two lateral ULt and one central UM) and lower (two lateral LLt and one central LM) anthers showed the same anatomical structure in all types of anthers. Furthermore, the cytological studies indicated typical karyokinesis, neutral-equatorial chondriokinesis, and successive cytokinesis taking place during microsporogenesis in all anthers of the androecium. As shown in our analyses, all *T. erecta* anthers formed morphologically uniform pollen grains. This rare feature in Commelinaceae species has been so far described only in *Aneilema lamuense* (Faden 1991), *Murdannia audreya*, *M. dimorphoides*, and *M. striatipeta* (Faden 2001), *Palisota* (Faden 1983, 1998), as well as *Plowmanianthus dressleri*, *P. grandifolius*, and *P. panamensis* (Hardy et al. 2004). In turn, most Commelinaceae species produce morphologically diverse pollen grains. For example, fertile lateral stamens of *Commelina benghalesis* and *Aneilema clarkei* have been shown to produce white pollen grains, while the pollen grains produced by the central stamen are yellow (Faden 2000). In the *Floscopa* and *Tinantia* genera, different coloured pollen grains are produced by upper and lower stamens. *T. anomala* produces white pollen grains in upper anthers and yellow pollen grains in lower anthers (Simpson et al. 1986; Gębura et al. 2014). In turn, *T. pringlei* produces cream-yellow and turquoise pollen grains in the upper and lower anthers, respectively (Faden 2000). *Tripogandra grandijlora* produces sterile boomerang-shaped pollen in long anthers and viable pollen grains typical of the Commelinaceae family in short anthers (Poole and Hunt 1980).

Additionally, the functional analysis of pollen grains formed in *T. erecta* revealed that morphologically undifferentiated pollen grains show the same viability and germinability. Therefore, it can be said that, despite the high morphological diversity of the *T. erecta* androecium, there are no staminodia and all stamens are functional. A frequently described strategy for attracting pollinators is the presence of staminodia in the androecium. Stamens transformed into staminodia do not produce pollen grains or produce sterile pollen. Staminodia are usually large and brightly coloured, which enhances the visual attractiveness of flowers to insects (Decraene and Smets 2001). These common structures in the family Commelinaceae (Table 1) have been described in over a dozen genera, e.g. *Aneilema*, *Cochliostema*, *Commelina*, *Murdannia*, *Palisota*, and *Tripogandra* (Faden 1992).

Therefore, based on the available literature and our observations, it can be claimed that species from the Commelinaceae family have all variants of differentiated androecium (Table 1). The stamens (1.) vary morphologically and produce morphologically identical pollen grains (*Tinantia erecta*, *Murdannia audreya*), (2.) vary morphologically and produce morphologically diverse pollen grains (*Tinantia anomala*), (3.) vary morphologically and functionally (staminodia) and produce morphologically identical pollen grains (*Aneilema lamuense*), (4.) vary morphologically and functionally and produce morphologically diverse pollen grains (*Commelina erecta*), (5.) vary morphologically and functionally and produce morphologically and functionally diverse pollen grains (*Commelina coelestis*, *C. dianthifolia*), (6.) vary morphologically and functionally and produce morphologically and functionally identical pollen grains (*Murdannia audreya*, *M. dimorphoides*, *M. striatipeta*, *Plowmanianthus dressleri*, *P. grandifolius*, *P. panamensis*), and (7.) do not vary morphologically and produce morphologically diverse pollen grains (*Aneilema neocaledonicum*). It should be emphasised that there are relatively few studies analysing the androecium in Commelinaceae with respect to the aforementioned traits (Table 1).

As we know, all Commelinaceae species are characterised by 1-day anthesis, which is not beneficial for entomophilous plants. We suggest that the heteromorphism of stamens is a special example of modification of the androecium increasing the visual attractiveness of flowers. The *T. erecta* analysed in this study, similarly to all Commelinaceae species, has morphologically different stamens, but importantly all produce viable and fertile pollen grains. Furthermore, our analyses have shown that *T. erecta* is also capable of self-pollination in addition to cross-pollination. It can therefore be concluded that the species uses its entire reproductive potential to ensure efficient pollination and, consequently, fertilisation, which increases its reproductive success.

## Conclusion

The present study was focused on comprehensive analysis of the *T. erecta* androecium, taking into account all morphological, anatomical, and cytological traits as well as the functionality of the pollen produced. These analyses showed that, despite their high morphological diversity, *T. erecta* stamens did not differ anatomically. Furthermore, the process of microsporogenesis followed by gametogenesis occurring in all the stamens yielded the same pollen grains in terms of morphology, cytology, and function. Thus, despite the large morphological diversity of the androecium, male gametophytes in this species are identical in every respect, which is a unique feature in species from the Commelinaceae family. Additionally, *T. erecta* is also capable of self-pollination. Therefore, it can be suggested that it uses its entire reproductive potential for efficient pollination and, consequently, sexual reproduction.
